# KADAM topical warm oxygen therapy device for diabetic foot ulcer-a novel approach

**DOI:** 10.1007/s40200-022-01172-3

**Published:** 2022-12-21

**Authors:** G. Arun Maiya, Megha Nataraj, Gagana K, Manjunatha Hande, Gabriel Sunil Rodrigues, Rajgopal Shenoy, Shiva S. Prasad

**Affiliations:** 1 Department of Physiotherapy, Centre for Diabetic Foot Care & Research (CDFCR), Manipal College of Health Professions (MCHP), Manipal Academy of Higher Education (MAHE), Manipal, 576104 Karnataka India; 2 Department of Medicine, Kasturba Medical College-Manipal, Manipal Academy of Higher Education (MAHE), Manipal, 576104 Karnataka India; 3 Department of Surgery, Kasturba Medical College-Manipal, Manipal Academy of Higher Education (MAHE), Manipal, 576104 Karnataka India

**Keywords:** Topical oxygen therapy, Wound healing, Diabetic foot ulcer, Ulcer management, Diabetes

## Abstract

**Purpose:**

Diabetic foot ulcer (DFU) is a significant healthcare burden demanding prompt attention. In the past decade, newer technologies such as topical oxygen therapy have grown increasingly popular. The purpose of the study was to determine effect of KADAM-a topical warm oxygen therapy (TWOT) medical device in healing of DFU.

**Methods:**

The KADAM medical device developed by Yostra Labs Pvt Ltd, delivered pure oxygen between 93 ± 3% concentration at an optimal temperature range of 39-42º Celsius to DFU wound site.

**Results:**

A total of 40 diabetic foot ulcer individuals, aged over 18 years were included in the study. Individuals with the Grade 1 DFU were 34 (85%), grade 2 were 5 (12.5%) and grade 3 were 1 (2.5%). The changes in initial area and final area for the various diabetic foot ulcer grades were as follows: 0.32 [0.12, 0.96] (< 0.001) for grade 1 ulcer, 0.76 [0.54, 1.17] (*P* = 0.013) for grade 2 ulcer and 1.26 for grade 3 ulcer. The percentage reduction in wound size achieved for the grade 1 & 2 DFU were 100%, and grade 3 was 75%.

**Conclusion:**

Topical warm oxygen therapy serves as an adjunctive modality to facilitate diabetic foot ulcer healing in the clinical practice.

## Introduction

A wide range of health-related implications are on the rise because of India becoming the world's epicentre for diabetes mellitus [1]. Diabetic foot ulcer (DFU) is one of the many consequences of diabetes mellitus and is a serious growing concern [2]. The burden of limb loss brought on by the fast-progressing nature of DFU significantly lowers the quality of life [3] of individuals affected by it thus increasing disability [4], economic or financial load [5], and dependency on caretakers. Additionally, the varying prevalence of DFU—6.38% [6] in South India versus 14.30% [7] in North India—indicates its widespread dispersion.

The presence of co-morbidities like hypertension, coronary artery disease, peripheral arterial or vascular disease, chronic kidney disease, obesity, addiction history to smoking, alcohol, or tobacco, long-standing diabetes history, uncontrolled glycemia, prior history of amputation, and altered autonomic system functioning raises the likelihood of developing a DFU [8]. Thus, early identification of an individual at risk for developing a diabetic foot ulcer may affect the timeliness of the prognosis for wound healing. This highlights the urgent requirement for strict measures and prompt follow-ups for diabetic foot care with medical specialists in suitable therapy selection to stop further progression of DFU among the affected individuals.

Topical oxygen therapy (TOT), a non-invasive treatment option, has been gaining popularity all over the world among the surgical and non-surgical management alternatives for DFUs. A recent systematic review by Nataraj et al., 2019 [9] demonstrated the efficiency of topical oxygen therapy in treating various grades of diabetic foot ulcers either entirely or by 50%. The use of TOT has been associated to minimal hazards, as opposed to the consequences of oxygen toxicity and barotrauma as shown with hyperbaric oxygen therapy (HBOT) [10], making TOT [11, 12] a recommended adjunctive therapy for DFU management.

The significance of topical oxygen and its various routes of application in the healing of chronic DFUs has previously been explored [9, 13–16]. The use of topical warm oxygen therapy has still not been investigated thoroughly. The mechanism of action of TWOT depends on the modifications brought about by warm oxygen [17] on the physical characteristics and malleability of the various structures of the integumentary system through an ideal rise in temperature and sufficient oxygenation to the tissues. The presence of oxygen also affects alterations in the enzymatic reactions at the wound bed [18]. Enzymatic activity increases linearly with every 0.1 °C increase in temperature [19]. However, denaturation of most animal or human body enzymes is seen when chemical bonds composed of amino acids or peptides break at temperatures exceeding 40 degrees Celsius (or 104-degree Fahrenheit).

The objective of the study was to evaluate KADAM, a topical warm oxygen therapy (TWOT) medical device, for the management of diabetic ulcers (DFUs).

## Materials & methods

### Ethical clearance

The study was approved by the Institutional Ethics Committee of Kasturba Hospital (IEC 506/2019) and Clinical Trial Registry India (CTRI/2020/01/023004). The study was conducted at the Centre for Diabetic Foot Care & Research (CDFCR) unit of Department of Physiotherapy of Kasturba Hospital, Manipal Academy of Higher Education, Karnataka, India.

### Study participants & study setting

The inclusion criteria were participant above 18 years, with a known diagnosis of type 2 diabetes mellitus, presence of a chronic non-healing diabetic foot ulcer which had previously failed to heal with standard care or other treatment modalities. The diabetic foot ulcers graded 1–4 on the Wagner’s Classification System were included. The exclusion criteria were age below 18 years, presence of foot ulcers of other (ischemic/neuro-ischemic) or unknown pathological condition, Wagner’s Grade 5 DFU, or ulcer location other than foot or ankle region, individuals referred for any surgical intervention other than debridement as a management for DFU.

The study participants were screened at inpatient and outpatient basis at the Department of Surgery and the Centre for Diabetic Foot Care and Research (CDFCR) unit in Kasturba Hospital, Manipal. Individuals who met the study eligibility and consented for participation provided a written approval for receiving the intervention using TWOT.

The demographic characteristics of participants are represented in Table 1.

**Table 1:** Represents the demographic characters of study participants (*N* = 40)

Characteristic	Mean (SD)	Shapiro Wilk P value
Age (years)	58.5 (9.81)	0.328
Height (cms)	164 (9.44)	< 0.001
Weight (kgs)	69.1 (14.8)	< 0.001
Body Mass Index (kg/m^2^)	25.4 (4.28)	0.146
Fasting Blood Glucose (mg/dL)	140 (31.7)	0.023
Duration of Type 2 Diabetes Mellitus (years)	13.0 (6.29)	0.294
Pulse Rate (bpm)	79.5 (7.37)	0.343
Oxygen Saturation (%)	99 (0.846)	< 0.001
Systolic Blood Pressure (mm Hg)	140 (10.7)	0.102
Diastolic Blood Pressure (mm Hg)	83.8 (9.06)	0.381
Ankle Brachial Index	1.09 (0.09)	0.059
Monofilament (out of 6)	1.68 (0.859)	< 0.001
Vibration Perception Threshold (volts)	40.6 (4.67)	0.642
	Number or Counts	Percentage
Gender:		
Female	4/40	10%
Male	36/40	90%

### Diabetic foot evaluation

The bilateral foot of the participants was closely observed by the qualified physiotherapists for the presence of any dryness of skin, alteration in skin colour, infection, callus, fissures, ingrown nails, prominent meta-tarsal head and swelling or oedema. A detailed bilateral foot examination was performed through assessment of the temperature on dorsal and plantar aspect using an infrared thermometer, presence of any altered sensations was assessed using cotton plug, pedal pulses of feet were assessed manually through palpation of the Tibialis posterior and Dorsalis pedis pulse, changes in ankle reflex response were assessed using reflex hammer. The loss of protective sensation (LOPs) was assessed with the participant in long-sitting position on assessment plinth using the Semmes–Weinstein 5.07 (10 g) Monofilament and Vibration perception threshold using the Neuro Touch ™ device. The product information available at [https://kodymedical.com/product/monofilament/] [www.neurotouch.co]. Specific areas in the plantar aspect of the feet were assessed as per user manual instructions and participants response to test was documented. The strength of lower extremity muscle groups was evaluated using Manual Muscle Testing Procedure [20].

### Ulcer evaluation

Prior to the delivery of intervention, the diabetic foot ulcer of the participants was cleaned thoroughly using normal saline 500 ml and Betadine-100 ml povidone-iodine solution intraperitoneal injection (IP) 10% weight per weight (w/w). The initial ulcer area was measured by the study investigator in length (centimetres) and width (centimetres) using an A4 size sterile transparent overhead projector (OHP) sheet for every treatment session of the participant without making any contact with ulcer site. The ulcers were identified as neuropathic and thereafter graded based on the Wagner’s Classification system.

### Topical warm oxygen therapy (TWOT) device

**Fig. 1:**
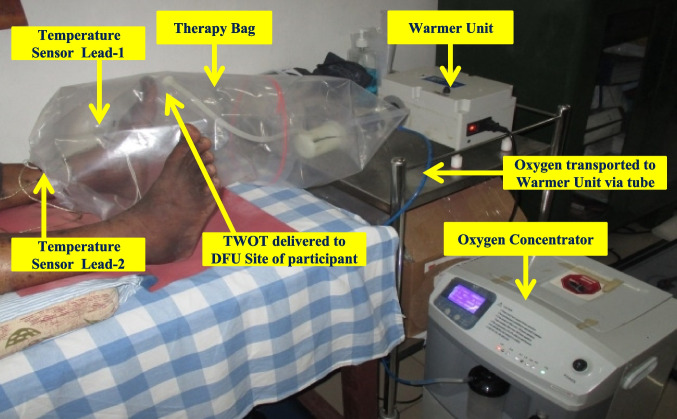
Participant is in the supine position on treatment plinth. The KADAM medical device has been used to deliver the Topical Warm Oxygen Therapy (TWOT) for a study participant with left diabetic foot ulcer (DFU) on plantar aspect

The topical warm oxygen therapy (TWOT) medical device used was KADAM designed by YOSTRA Labs Private Ltd, Bangalore, India. The device comprised of an oxygen concentrator which delivered pure oxygen between 93 ± 3% concentration, a warmer unit with temperature sensors that warmed the air and oxygen and maintained an optimal internal environment inside the therapy bag at desired level between 39-42º Celsius. The participants were asked to lie down in supine position on the treatment plinth. The large disposable therapy bag accommodated the entire involved ulcer foot leg of the participant to be placed for the intervention. One end of therapy bag was connected to the oxygen diffuser inlet from where the oxygen delivery probe was attached to the smaller disposable ulcer therapy bag. Both the therapy bags were fastened using an elastic band and stopper to avoid any leakage of air and oxygen to the outer environment as shown in Fig. 1.

### Outcome assessment & follow-up

The diabetic foot ulcer area was observed on all days of the intervention for any changes in the formation of granulation tissue, epithelialization and mean ulcer healing time till the complete wound closure were achieved. Sequential photographs and measurement of the wound were taken on treatment days regularly until the wound closure was observed. The primary outcome measure was the rate of wound contraction (ROC). The numbers of sessions of topical warm oxygen therapy delivered throughout the wound healing period were recorded for every study participant by the investigator(s). The ulcer area was dressed after the TWOT intervention daily using Megaheal-15 g amorphous hydrogel wound dressing with colloidal silver and Silver Nitrate gel 0.2% weight per weight (w/w) 20 g. The study participants were also prescribed individualized footwear modification with offloading as indicated from the Artificial Limb Centre (ALC) Department in Kasturba Hospital.

### Therapeutic dosage of topical warm oxygen therapy (TWOT)

**Fig. 2:**
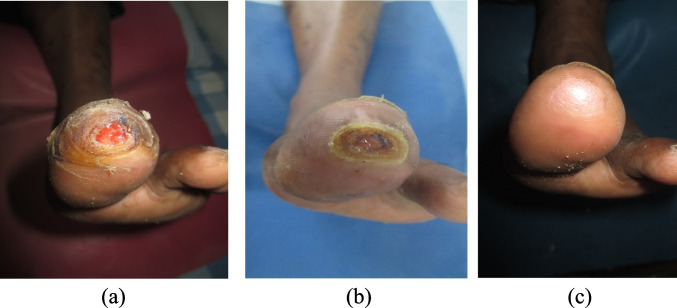
Image (**a**) shows inflammatory phase of study participant with a Grade 1 Diabetic Foot Ulcer (DFU); Image (**b**) shows DFU in proliferative phase & Image (**c**) shows complete DFU wound closure with delivery of topical warm oxygen therapy (TWOT) therapy with good skin health & no scar formation

The details of the therapeutic dosages for the delivery of topical warm oxygen therapy for each study participant are represented in the Table 2. The stages of wound healing achieved in one of the study participants with DFU is depicted in Fig. 2.

**Table 2:** Represents therapeutic dosage of TWOT intervention

Parameters	Inflammatory Phase	Proliferative Phase	Maturation/healing Phase
Duration of WOT treatment (mins)	45 min	30 min	30 min
Frequency of treatment (days/week):	5–6 days/week	3 days/week	2 days/week

## Statistical analysis

The demographic characteristics of study participants like age (years), body composition (height, weight, body mass index in kilogram/meter square), glycaemic values (fasting blood sugar in milligram/decilitre & glycated haemoglobin as percentage), blood pressure (millimetres of mercury), loss of protective sensation both monofilament & vibration perception threshold testing (LoPs) were analysed using descriptive statistics in Jamovi Software [21, 22]. Their values have been represented as mean & standard deviation in Table 1. The different grades of Wagner’s classification of diabetic foot ulcers were reported as a percentage score in Table 1. The dimensions of ulcer area were recorded as length X width (centimetre square) for the grade 1, grade 2 and grade 3 DFUs till complete wound closure. The initial area (IA) of DFU comprised of length X width measurement on initial day of assessment. The final area (FA) of DFU comprised of length X width measurement on final day when the wound closure was observed. The difference in initial area and final area of DFU was calculated. The initial area of DFU versus final area with wound closure are represented as median and inter quartile range in Table 3. The rate of wound contraction (ROC) was calculated and thereafter represented as median and interquartile range in Table 3. The wound healing achieved across the different grades of DFUs is represented as percentage values. The level of significance was set at P value ≤ 0.05.

**Table 3:** Represents the ulcer area and ROC of 40 DFU participants

Ulcer Grade	Ulcer Area	Median [IQR]	Shapiro Wilk P value
Grade 1 DFU	Initial Area (cms)	0.49 [0.14, 1.37]	< 0.001
	Final Area (cms)	0.00 [0.00, 0.23]	< 0.001
	ROC (cm^2^)	0.01 [0.00, 0.03]	< 0.001**
Grade 2 DFU	Initial Area (cms)	1.50 [0.78, 1.53]	0.037
	Final Area (cms)	0.06 [0.02, 0.36]	0.024
	ROC (cm^2^)	0.01 [0.01, 0.05]	0.214
Grade 3 DFU	Initial Area (cms)	1.26 [1.26, 1.26]	None
	Final Area (cms)	0.00 [0.00, 0.00]	None
	ROC (cm^2^)	0.02 [0.02,0.02]	None

## Results

A total of 40 participants with chronic diabetic foot ulcers participated in the study. The demographic characteristics of participants are represented as mean and standard deviation, shown in Table 1. The diabetic foot ulcers were classified according to Wagner’s System and represented as frequency-percentage value.

In this study majority of study participants were males. Furthermore, the number of grade 1 diabetic foot ulcers were 34 (85%), grade 2 were 5 (12.5%) and grade 3 were 1 (2.5%). All participants had one neuropathic type of diabetic foot ulcer located on plantar/dorsal aspect of right/left foot. All participants had an altered monofilament and vibration perception threshold on their affected extremity on comparison to non-affected extremity. The duration of diabetic foot ulcer was 0.772 [0.4, 0.85] years (*P* < 0.001).

The initial and final areas of different diabetic foot ulcer grades are represented in Table 3 as median and interquartile range. The changes in initial area and final area for the various diabetic foot ulcer grades were as follows: 0.32 [0.12, 0.96] (*P* < 0.001) for grade 1 ulcer, 0.76 [0.54, 1.17] (*P* = 0.013) for grade 2 ulcer and 1.26 [1.26, 1.26] for grade 3 ulcer. The percentage reduction in wound size achieved for the grade 1 diabetic foot ulcer were between 85–100%, 50–75% for grade 2 ulcer and 100% for grade 3 ulcer. The average sessions of topical warm oxygen therapy required in wound closure were as follows: 16.17 sessions for grade 1 ulcer, 23 sessions for grade 2 ulcer and 17 sessions for one participant with grade 3 ulcer.

## Discussion

The objective of the study was to evaluate KADAM, a topical warm oxygen therapy (TWOT) medical device, for the management of diabetic ulcers (DFUs). The study observed a significant reduction in ulcer size across grade 1, 2, 3 DFUs with the topical warm oxygen therapy intervention.

In the present study topical warm oxygen therapy intervention was delivered between optimal temperatures 39–42 degree centigrade which is partially above the normal body temperature of 36.1 to 37.2 degree centigrade [97-to-99-degree Fahrenheit]. The small change in temperature of oxygen in addition to the warmth delivered may have promoted the micro-cellular level changes at the ulcer site through improved diffusion of oxygen [23].

The DFUs of different grades 1, 2 and 3 in the study showed complete healing with full wound closure. However, in previous studies that used topical continuous oxygen therapy alone without warmth, marginal reduction was observed in the diabetic foot ulcer size [11–16, 24–27]. This suggests that addition of warmth to continuous delivery of topical oxygen, enhances the healing capacity of wound through an alteration in the wound healing mechanism.

Adjunctive cyclical pressurised TWO2 therapy outperformed optimum standard of care alone in treating chronic DFUs at both 12 weeks and 12 months, according to randomized controlled trial study by Frykberg RG et al., 2020 [11]. The OTONAL trail by Tang T.Y et al., 2021 observed wound closure of more than 75% in 70% of its study participants. Reduction in wound area by 3 months was 91.3% with mean time for complete closure being 77.6 $\pm$ 32.5 days [15]. This difference in mean time for 100% closure was much higher when compared to the present study [15]. The present study delivered fewer sessions and obtained similar results in wound closure. Another study by Anirudh V et al., 2021 used KADAM™ topical controlled warm oxygen therapy device and observed a significant reduction in ulcer area between intervention and standard care group [16]. These findings are suggestive of effectiveness of the topical warm oxygen therapy intervention in the healing dynamics of chronic non-healing diabetic foot ulcers.

It is possible that warmth increases the vasodilatation which results in maintenance of optimal hydration and humidification levels of diabetic foot ulcer site. Furthermore, it results in adequate oxygen and nutritional supplementation which facilitates improved wound healing capacity. [11, 17, 28]. It provides an up-regulated wound micro-environment [9, 29] that aids healing of DFUs through improved cellular metabolism, improved enzymatic rate of reaction at both wound and peri-wound area. It also facilitates improved collagen synthesis by catalysing the enzymatic reactions which utilize molecular oxygen namely hydroxylase, lysyl hydroxylase, and lysyl oxidase [30].

The overall rate of wound healing in other topical oxygen delivery application modes [9] suggests a 100% healing for smaller DFU of grade 1, while DFUs of grade 2 and above showed reduction in size by 50% with changes in wound depth and tissue margins.

### Strength of this study

- The study findings aid in understanding the role of topical warm oxygen therapy for healing of diabetic foot ulcers across different grades.
- There were no severe adverse effects reported during & after utilization of TWOT among study participants.
- The study found topical warm oxygen therapy to be a simple yet safe adjunctive modality for diabetic wound care in routine hospital/clinical practice/podiatry settings.
- A pre-clinical study on Wistar rats was conducted by the study authors and found to be effective in improving healing dynamics of diabetic foot ulcers.

### Limitations of this study

- The study did not include the assessment of the pH of skin and wound area, transcutaneous oxygen measurement (TCOM) and changes in inflammatory biomarkers across the various phases of wound healing.
- Future studies including these aspects of diabetic foot ulcer assessment may provide further insight to the role of topical warm oxygen therapy.

## Conclusion

Topical warm oxygen therapy serves as an adjunctive modality to facilitate diabetic foot ulcer healing in clinical practice.

## Data Availability

The availability of study data will be provided on reasonable request made to the corresponding author.
